# Supplementary material for: Poly(rC)-Binding Protein 1 Limits Hepatitis C Virus Virion Assembly and Secretion
Source: Viruses. 2022 Jan 29;14(2):291. doi: 10.3390/v14020291 (PMC8877974; doi:10.3390/v14020291)
Supplement: Supplementary file 1 [file viruses-14-00291-s001.zip › viruses-1571447-supplementary.pdf]

SUPPLEMENTARY INFORMATION

SUPPLEMENTARY METHODS

Plasmids

To generate the pPCBP1(N)FLAG-puro plasmid, the complete PCBP1 coding sequence was amplified using PCBP1noAUG-KpnI-FW (5'-AAG CTG GCG GTA CCG GAG ATG CCG GTG TGA CTG-3') and PCBP1-XhoI-RV (5'-TTT TTC TC GAG CTA GCT GCA CCC CAT GCC CTT -3') and the high-fidelity Hot Start Q5 DNA polymerase (NEB). The PCR product was digested with KpnI and XhoI and ligated in a pcDNA3.1(+)-derived plasmid that contained a 3xFLAG tag located downstream of a human cytomegalovirus (CMV) immediate early enhancer/promoter and upstream of a bovine growth hormone (bGH) polyadenylation signal; this insertion left the PCBP1 coding sequence in frame with the upstream 3x FLAG tag. To note, this pcDNA3.1(+)-derived vector also contained a puromycin resistance gene located downstream of a phosphoglycerate kinase 1 (PGK) promoter, and upstream of a simian virus 40 (SV40) polyadenylation signal.

To generate the pFLuc-puro plasmid, the complete Firefly luciferase coding sequence was amplified using FLuc-NheI-FW (5'-ATC CGC TAG CAT GGA TTA CAA GGA C-3') and FLuc-XhoI-RV (5'-AAG GTA TCT CGA GTT AGT AAA CAA GAT AAT TGC TCC TAA AGT A-3') using the high-fidelity Hot Start Q5 DNA polymerase (NEB). The PCR product was digested with NheI and XhoI and ligated into the same pcDNA3.1(+)-derived plasmid, between the CMV enhancer/promoter and the bGH polyadenylation signal; this insertion removed the 3xFLAG tag.

The pJ6/JFH1 FL RLuc WT ("RLuc-wt") viral sequence contains include a *Renilla* luciferase (RLuc) reporter gene inserted between p7 and NS2 [1]. The pJ6/JFH-1 FL RLuc-NS5A-GFP ("NS5A-GFP") plasmid contains a GFP insertion between P2390 and L2391 within the NS5A domain III, as previously described [2]. The NS5A-GFP region was subcloned into the pJ6/JFH-1 FL RLuc WT backbone using the *AvrII* and *XbaI* restriction sites.

To construct a JFH-1_T-RLuc viral genome template, a MluI site was created between the p7 and NS2 genes by PCR amplification of the pJFH-1_T plasmid using JFH1-p7NS2-MluI-FW (5'-CCT ATG ACG CGT CTG TGC ACG GAC AGA TAG GC-3'), JFH1-p7NS2-MluI-RV (5'-CAG ACG CGT CAT AGG CAT AAG CCT GCC G-3'), and the high-fidelity Hot Start Q5 DNA polymerase (NEB). The PCR product was digested with MluI and dephosphorylated with calf intestinal phosphatase (Quick CIP, NEB) and ligated with an insert containing a *Renilla* luciferase (RLuc) with a C-terminal foot-and-mouth disease virus 2A peptide (FMDV2A), which had been cut out of the pJ6/JFH WT RLuc plasmid by MluI digestion. The correct orientation of the insert was screened by digestion with BstBI and NotI and by sequencing. Once a plasmid with the correct insert orientation was identified, the E1 through NS2 region was cut out using the BsiWI and NotI unique cut sites and ligated into the original pJFH-1_T plasmid backbone. The inserted E1 through NS2 region was sequenced to verify that the PCR amplifications had not introduced any unexpected substitutions.

Puromycin selection of cells and infection with JFH-1_T

To select cells that would stably express PCBP1(N)FLAG or the FLuc control protein, 15-cm dishes were seeded with 2×10^6 Huh-7.5 cells one day prior to transfection with 36 μg of pPCBP1(N)FLAG-puro or pFLuc-puro plasmid and 30 μL Lipofectamine 2000 diluted in 12 mL

Opti-MEM serum-free media (Thermo Fisher Scientific). Two days post-transfection, the media on all transfected plates and one non-transfected plate was changed for complete Huh-7.5 media supplemented with 3 µg/mL puromycin (Thermo Fisher Scientific), which was replenished every two to three days, passaging the cells as necessary. Once all cells in the non-transfected plate were completely dead (within 7 days of selection), selected cells could be used for infection experiments. Selected cells were transfected with siRNA duplexes and infected with JFH-1_T at an MOI of 0.05 as described in the article's main methods; the selection pressure of 3 µg/mL puromycin was maintained in these cells' media until their infection with JFH-1_T, after which they were kept in puromycin-free media.

REFERENCES

1. **Pietschmann T**, **Kaul A**, **Koutsoudakis G**, **Shavinskaya A**, **Kallis S**, **Steinmann E**, **Abid K**, **Negro F**, **Dreux M**, **Cosset F-L**, **Bartenschlager R**. 2006. Construction and characterization of infectious intragenotypic and intergenotypic hepatitis C virus chimeras. PNAS **103**:7408–7413.

2. **Moradpour D**, **Evans MJ**, **Gosert R**, **Yuan Z**, **Blum HE**, **Goff SP**, **Lindenbach BD**, **Rice CM**. 2004. Insertion of green fluorescent protein into nonstructural protein 5A allows direct visualization of functional hepatitis C virus replication complexes. Journal of Virology **78**:7400–7409.

SUPPLEMENTARY FIGURES

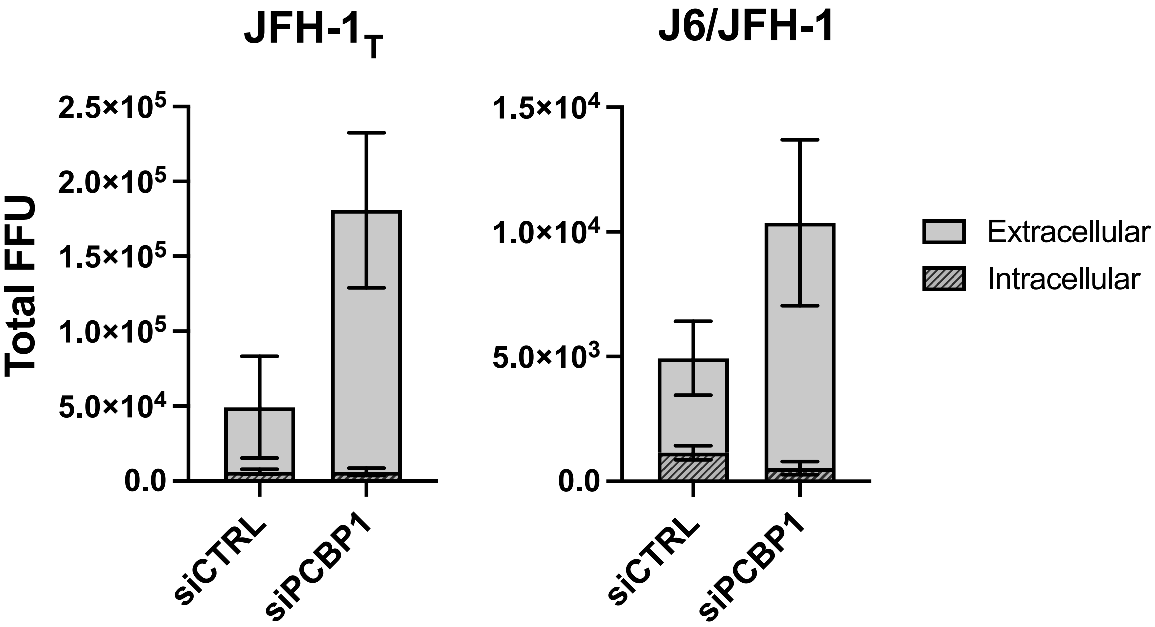

Supplementary Figure S1. PCBP1 knockdown increases the total quantity of infectious virions produced by the JFH-1$_T$ and J6/JFH-1 strains. To calculate the total quantity of infectious virions (FFU) present in a sample, the viral titer (FFU/mL) was multiplied by the total volume of the sample (mL). These data were derived from the same experiments presented in Figure 1 and are representative of three independent biological replicates. Error bars represent the standard deviation of the mean.

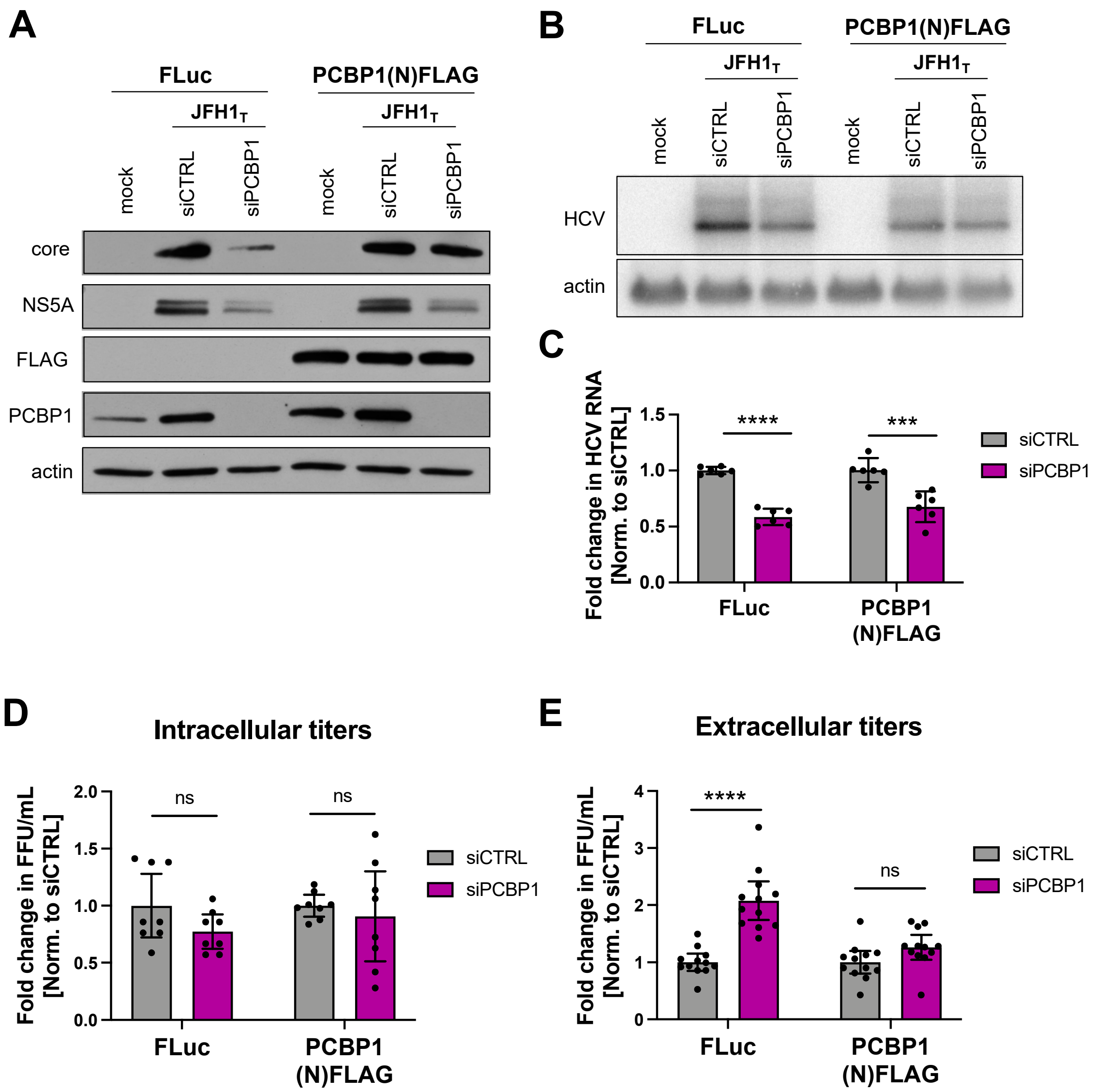

Supplementary Figure S2. Ectopic PCBP1 expression can reduce the impact of endogenous PCBP1 knockdown on extracellular viral titers. Huh-7.5 cells that were puromycin-selected to stably express ectopic Firefly luciferase (FLuc, control) or PCBP1 with a N-terminal FLAG tag (PCBP1(N)FLAG) were transfected with siCTRL or siPCBP1 two days prior to infection with JFH-1T (MOI 0.05). Three days post-infection, total intracellular protein, RNA, and intracellular and extracellular virus were collected. **(A)** Viral protein expression analysis by Western blot. **(B)** Viral RNA accumulation analysis by Northern blot and **(C)** quantification by RT-qPCR. **(D)** Intracellular and **(E)** extracellular (secreted) virus titers, quantified by FFU assay. For (**C**–**E**), data from each experiment was normalized to the mean of the cell-matched siCTRL condition before replicate data were combined. All data are representative of three independent biological replicates, with the exception of (**B**) which is an N = 1. Error bars represent the standard deviation of the mean. Statistical significance was calculated by paired t-test (ns, not significant; *** $p < 0.001$; **** $p < 0.0001$).

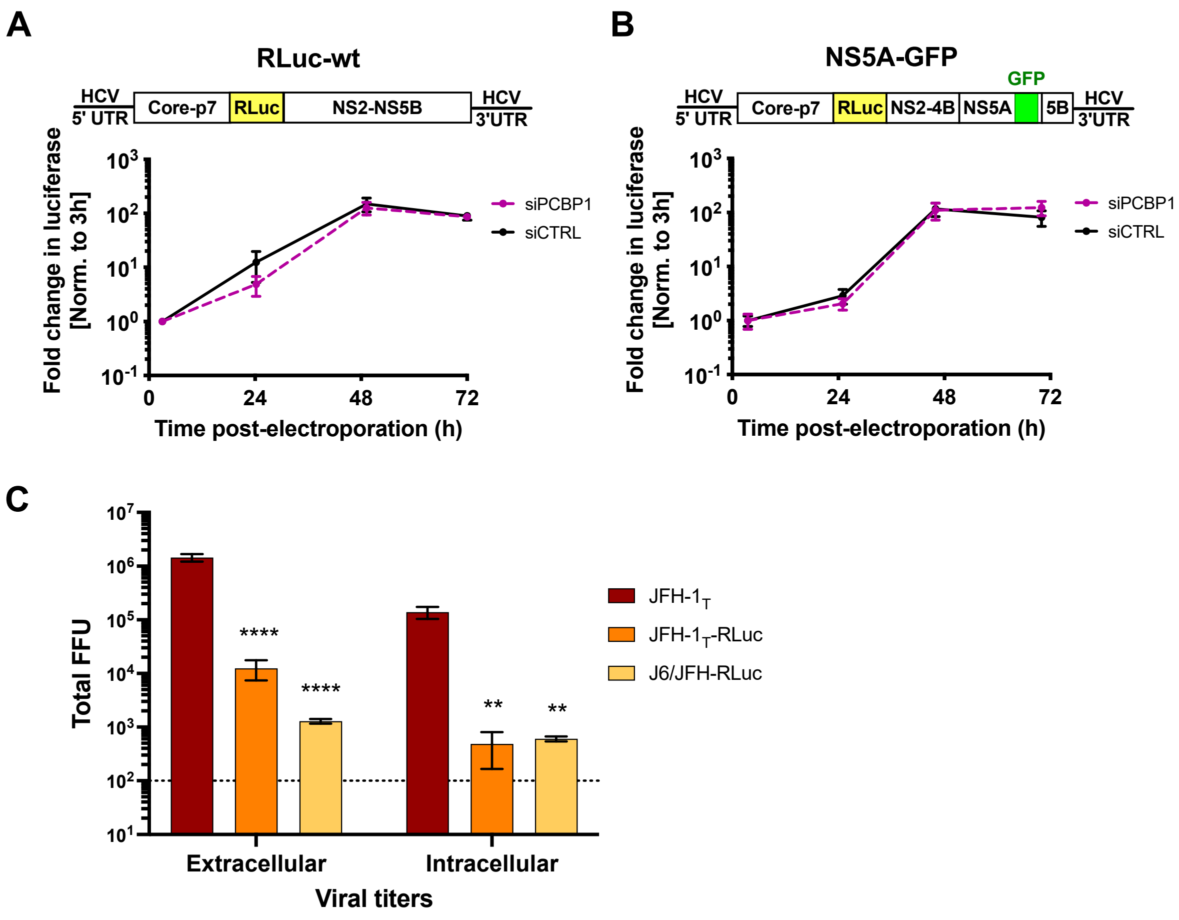

Supplementary Figure S3. PCBP1 has no effect on RNA replication in the absence of efficient virion assembly, and the addition of a RLuc gene reduces the assembly efficiency of HCV genomes. SiRNA-transfected Huh-7.5 cells were electroporated with 5 µg of (**A**) full-length J6/JFH RLuc WT RNA, or of (**B**) a full-length J6/JFH RLuc with a GFP insertion in the NS5A gene (NS5A-GFP), which had previously been shown to impair virion assembly without impairing viral RNA replication. Luciferase activity was monitored for three days post-electroporation, and RLuc values were normalized to the early timepoint (3h) to control for disparities in electroporation efficiency between experiments. (**C**) Equal amounts (10 µg) of JFH-1_T, JFH-1_T-RLuc, or J6/JFH-RLuc RNAs were electroporated into Huh-7.5 cells; intracellular and extracellular viruses were collected three days post-electroporation and titered by focus-forming unit assay. Compared with the untagged JFH-1_T RNA electroporations, the extracellular titers were reduced by over 100-fold and 1000-fold for JFH1_T-RLuc and J6/JFH-RLuc, respectively; intracellular titers were reduced by more than 200-fold for both luciferase reporter genomes. Data in (**A**) and (**B**) are representative of three independent biological replicates; data in (**C**) is representative of two independent biological replicates, and the limit of detection is indicated. Error bars represent the standard deviation of the mean. Statistical significance was calculated by t-test (** $p <$ 0.005, **** $p < 0.0001$).

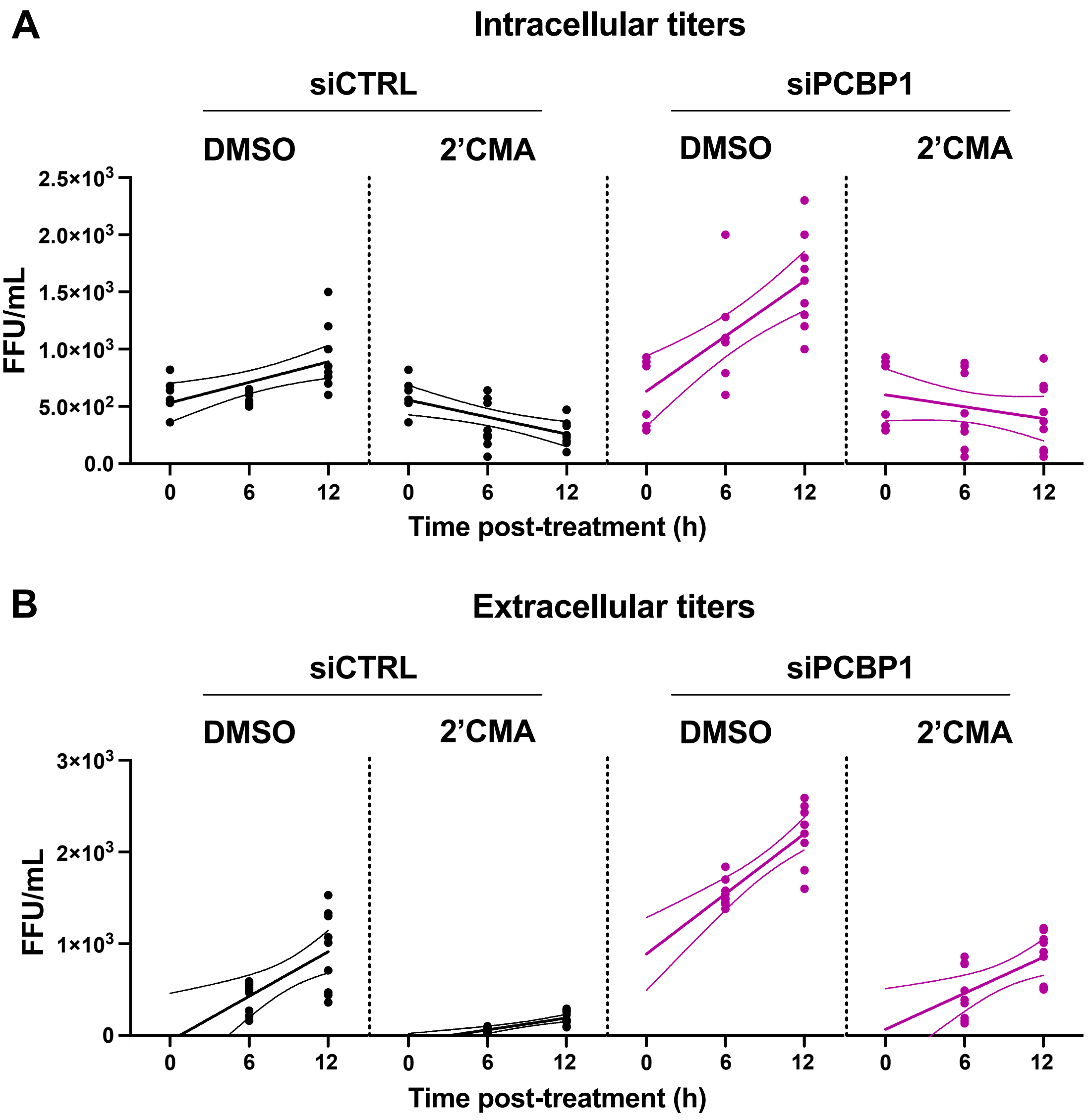

Supplementary Figure S4. Linear regressions to calculate the rates of intracellular virion accumulation and of virion secretion after 2'CMA treatment. (A) Intracellular and (**B**) extracellular viral titers were collected during a 12-hour window after the infected cell culture medium was replaced with media containing 2'CMA or vehicle (DMSO). Solid lines show the simple linear regression calculated using the least squares method for each experimental condition; the slope (and error of the slope), in FFU/h, are reported as the virion accumulation rate and virion secretion rates found in Figure S3D, F. The dotted lines mark the 95% confidence interval of the regression. Each dot represents a technical replicate, and three independent biological replicates were pooled together.
